# Production of Fluorescent Dissolved Organic Matter by Microalgae Strains from the Ob and Yenisei Gulfs (Siberia)

**DOI:** 10.3390/plants11233361

**Published:** 2022-12-03

**Authors:** Nikolay V. Lobus, Anton M. Glushchenko, Alexander A. Osadchiev, Yevhen I. Maltsev, Dmitry A. Kapustin, Olga P. Konovalova, Maxim S. Kulikovskiy, Ivan N. Krylov, Anastasia N. Drozdova

**Affiliations:** 1 Timiryazev Institute of Plant Physiology, Russian Academy of Sciences, Botanicheskaya Street 35, 127276 Moscow, Russia; 2 Shirshov Institute of Oceanology, Russian Academy of Sciences, Nakhimovskiy Prospect 36, 117997 Moscow, Russia; 3 Marine Research Center at Lomonosov Moscow State University, Leninskie Gory 1, 119992 Moscow, Russia; 4 Department of Chemistry, Lomonosov Moscow State University, Leninskie Gory 1 bldg. 3, 119234 Moscow, Russia

**Keywords:** Arctic, algae, morphology, molecular biology, biogeochemical cycles, dissolved organic matter, fluorescence, PARAFAC

## Abstract

Dissolved organic matter (DOM) is an important component of aquatic environments; it plays a key role in the biogeochemical cycles of many chemical elements. Using excitation–emission matrix fluorescence spectroscopy, we examined the fluorescent fraction of DOM (FDOM) produced at the stationary phase of growth of five strains of microalgae sampled and isolated from the Ob and Yenisei gulfs. Based on the morphological and molecular descriptions, the strains were identified as diatoms (*Asterionella formosa*, *Fragilaria* cf. *crotonensis*, and *Stephanodiscus hantzschii*), green microalgae (*Desmodesmus armatus*), and yellow-green microalgae (*Tribonema* cf. *minus*). Three fluorescent components were validated in parallel factor analysis (PARAFAC): one of them was characterized by protein-like fluorescence (similar to peak T), two others, by humic-like fluorescence (peaks A and C). The portion of fluorescence intensity of humic compounds (peak A) to the total fluorescence intensity was the lowest (27 ± 5%) and showed little variation between species. Protein-like fluorescence was most intense (45 ± 16%), but along with humic-like fluorescence with emission maximum at 470 nm (28 ± 14%), varied considerably for different algae strains. The direct optical investigation of FDOM produced during the cultivation of the studied algae strains confirms the possibility of autochthonous production of humic-like FDOM in the Arctic shelf regions.

## 1. Introduction

Algae are a key part of aquatic ecosystems, and aquatic life depends directly or indirectly on their activity [1]. Being the most important contributors to primary production in the world ocean, they form the base of the food web and affect biogeochemical cycles of many chemical elements [2,3,4]. In particular, dissolved primary production released through passive diffusion and active exudation processes [5] as well as organic matter liberating from decaying detritus represent an integral part of dissolved organic matter (DOM), the second largest bioavailable carbon pool in the ocean after the dissolved inorganic carbon pool [6,7]. DOM affects carbon dynamics, nutrient availability, microbial growth, etc. [8]. Additionally, the chromophoric fraction of DOM (CDOM) diminishes the negative effects of ultraviolet radiation on plankton populations [9], regulates the penetration of light into the water column [10], and may have a significant impact on the water color [11].

Ecosystems are currently undergoing significant transformations in response to changing environmental conditions caused by global warming and increasing anthropogenic pressure [12]. Studying the climate change impact on the environment and of the mechanisms of ecosystem response, forecasting further consequences of these processes, and developing environmental monitoring programs are nowadays receiving much attention [13]. In this regard, algae are widely studied for several important issues: (1) as a source of potential risk for the aquatic ecosystems due to harmful algae blooms that are reported to have been more frequent, intense, and widespread in the past decades [14]; (2) as the base of food web having enormous implications for the higher trophic levels [15]; (3) as organisms that can increase carbon assimilation under elevated CO_2_ [16,17]; and (4) as important indicators of the environmental situation and thus a significant component of various monitoring programs for assessing water quality [18]. Recently, it was suggested that plankton organisms are even more sensitive indicators of changes than the abiotic environmental variables because nonlinear responses of biological communities may amplify weak environmental shifts [15,19]. Another important issue is considering the algae as a potential source for green and blue technologies and for manufacturing. Nowadays, algae receive great attention due to their potential for resolving a list of contemporary concerns, such as CO_2_ emissions, alternative energy, water purification, and bioremediation [16].

The Arctic Ocean is among the regions most affected by the ongoing climate change, resulting in increases in air temperature and decreases in ice coverage during the warm season. These changes induce significant modification of local ecosystems, for example, increasing both the vegetation period and river discharges. The major Arctic rivers have been the focus of numerous studies over the past decades, which have shown that these rivers are important sources of DOM for the Arctic Ocean, and knowledge of DOM sources is essential for predicting the impact of climate change on marine ecosystems [20].

Optical studies of CDOM are widely applied in studies of carbon cycling in the Arctic since (i) spectroscopic techniques present various benefits in term of the high sensitivity, easy and rapid analysis, and low cost [21]; and (ii) a large amount of accumulated data makes it possible to trace the interannual and spatial variability of the CDOM quality and its distribution. One of the main shortcomings of optical approaches is that the interpretation of the optical data is not straightforward: CDOM with similar spectral characteristics can be formed during various biogeochemical processes. Therefore, the studies of CDOM supplied by individual ecosystem components are widely demanded. In particular, the quality of CDOM released during vital activity of Arctic algae species is essential for both the study of their interactions with the environment and for the development of ecological monitoring programs in the region [22].

The present study aims to characterize the quality of DOM formed as a result of intravital and postmortem algae excretion as well as DOM transformation during cultivation of individual microalgae strains typical for the Ob and Yenisei rivers. We focus on PARAFAC decomposition of excitation-emission matrices (EEMs) of FDOM to evaluate the set of individual fluorescent components that can be produced in natural environments. We expect that laboratory experiments on microalgae strain cultivation may improve the interpretation of results of field investigations, thereby providing more reliable information on DOM sources in the Arctic. This research topic was selected for the following reasons:

(1) It has been demonstrated that some algae cultures contribute significant free carbohydrates only during late bloom conditions when algae are abundant and under physiological circumstances resembling stationary or declining culture [23]. Under such conditions additional CDOM can also be released so in this study we focus on CDOM at the stationary phase of algae growth. The cultivation was interrupted when the cultures entered the stationary phase.
(2) A broad spectrum of optical methods is available for assessing CDOM parameters, from relatively simple measurements of CDOM UV adsorption to more advanced techniques such as excitation-emission matrix (EEM) fluorescence with PARAFAC decomposition [11,21,24].
(3) CDOM of algae origin has been studied using the optical indices calculated from absorbance and fluorescence spectra [25,26], analysis of synchronous fluorescence spectra [27] and EEMs by the conventional “peak picking” technique [28] and EEM fluorometry combined with PARAFAC [29,30,31,32]. The study of the fluorescence fraction of DOM (FDOM) produced during the cultivation of the arctic algal species will make a significant contribution to understanding the diversity of autochthonous FDOM in aquatic systems.
(4) Previously, it has been determined that CDOM is produced by live algae [28] and by bacteria using non-fluorescent organic matter derived from phytoplankton [33], as well as via phytoplankton degradation [30] and live algae excreting simple phenols that can be slowly transformed to humic substances [34]. Our study aims to characterize the bulk FDOM formed during cultivation of microalgae strains.
(5) In the last two decades, there has been a tendency to revise the systematic position of a large number of previously described species as well as to describe new microalgae species. The absence of comprehensive morphological and molecular genetic data in scientific papers creates difficulties for further verification and validation of the results. In this study we provide a comprehensive description of the algae strains to ensure both the accuracy of the definition of species and the reliability of data comparison in the future.

## 2. Results

### 2.1. Morphological Description of the Algae Strains

#### 2.1.1. Diatoms

The morphological description of diatoms was performed in accordance with [35,36,37,38].

***Asterionella formosa* Hassal (strain ARC01)**

**Figure 1:**
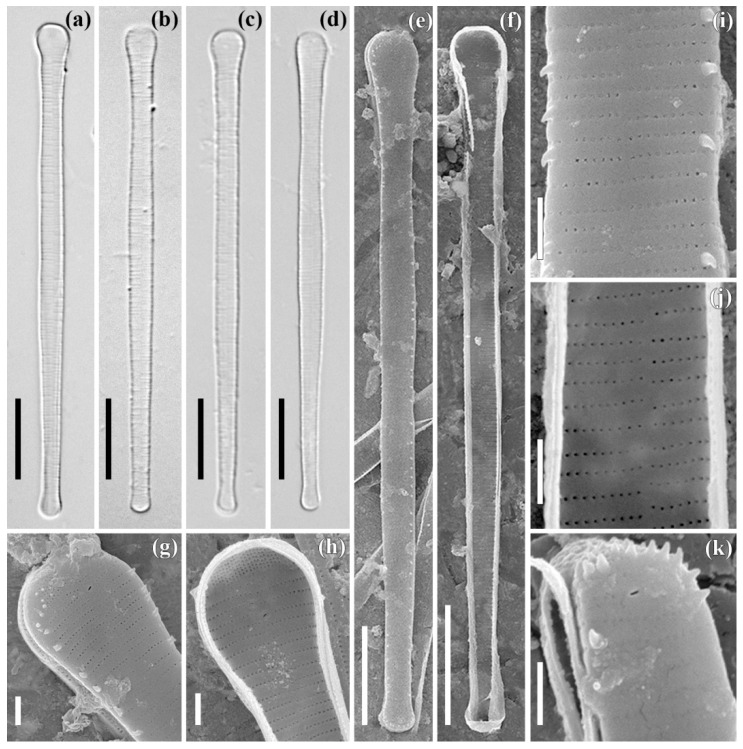
*Asterionella formosa* Hassal. Strain ARC01. Slide no. 08438. LM, DIC (**a**–**d**). SEM, external view (**e**,**g**,**i**,**k**), internal view (**f**,**h**,**j**). (**a**–**d**) size diminution series; (**e**,**f**) general view; (**g**,**h**) headpole; (**i**,**j**) central part; (**k**) footpole. Scale bars: 10 μm (**a**–**f**), 1 μm (**g**–**k**).

**The light microscopy** (LM) images are shown in Figure 1a–d. The valves are heteropolar and long with asymmetrical margins. The valves taper slightly near the footpole and are of length 59.5–61.3 μm and width 3.0–3.2 μm (center). The valve ends are of different shapes and sizes: the footpole is spatulate, 3.9–4.4 μm in width; and the headpole is small, sub-capitate, and 2.3–2.6 μm in width. The central sternum is very narrow and poorly visible. The striae are parallel and number 28–30 in 10 μm.

**The scanning electron microscopy** (SEM) external view is shown in Figure 1e,g,i,k. The valve face is flat (Figure 1e,g,i). The striae on both sides of the central sternum are unevenly spaced and comprised of small, rounded areolae (Figure 1g,i). The areolae are occluded (Figure 1i,k). Small claw-like (sometimes, flattened or conical) spines are located irregularly at the valve face/mantle junction between the striae (Figure 1e,g,i). Apical pore fields are present on both valve ends (Figure 1h,g,k). The openings of the rimoportulae are slit-like, differing from the openings of the areolae (Figure 1g,k).

**The SEM** internal view is shown in Figure 1f,h,j. The rimoportulae are oriented almost transversely at the valve ends. Usually, a rimoportula is present at both ends of the valve (see Figure 1g,k), but two rimoportulae at the “headpole” were observed in one valve (Figure 1h). The rimoportula lips are weakly expressed (Figure 1h). The apical pore field is separated from the striae. The apical pore field consists of 8–9 transverse rows formed by round pores, similar in shape and size to the areolae of the striae (Figure 1g,h). The striae are also unevenly spaced on both sides of the central sternum (Figure 1j). The specimens shown are slide no. 08438 and stub no. ARC 01 (oxidized culture strain ARC01, isolated from sample no. 3935 from the Gulf of Ob).

***Fragilaria* cf. *crotonensis* Kitton (strain ARC03)**

**Figure 2:**
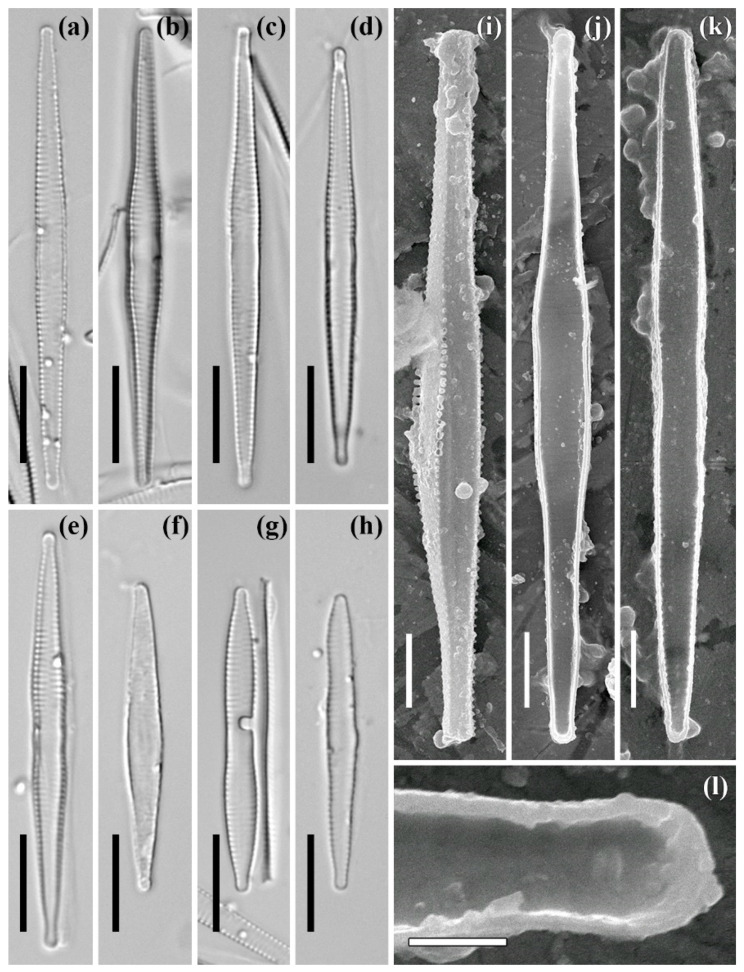
*Fragilaria* cf. *crotonensis* Kitton. Strain ARC03. Slide no. 08440. LM, DIC (**a**–**h**). SEM: (**i**) external view, (**j**–**l**) internal view. (**a**–**h**) size diminution series, (**i**–**k**) general view, (**g**,**h**) valve end. Scale bars: 10 μm (**a**–**h**), 5 μm (**i**–**k**), 1 μm (**l**).

**The LM** images are shown in Figure 2a–h. The valves are fusiform with a more or less swollen central part, which has a double undulation. The ends are capitate to sub-capitate. The dimensions are: length 30.4–47.8 μm, and width 3.1–3.7 μm (at the widest part). The axial area is narrow lanceolate, with clear fascia at the central area. The striae are parallel and number 17–18 in 10 μm.

**The SEM** external view is shown in Figure 2i. The valve face is weakly undulate due to raised interstriae (virgae). There are small flattened (in the central part of valve) or conical (closed to the ends) spines at the valve face/mantle junction, between the striae (Figure 2i).

**The SEM**, internal view is shown in Figure 2j–l. The central part of the valve varies in shape and width in different specimens (Figure 2j,k). The striae are noticeably narrower than the interstriae (Figure 2j,k). A single rimoportula is oriented parallel to the striae (Figure 2l). The rimoportula lips are well expressed (Figure 2l). The specimens shown are slide no. 08440, stub no. ARC 03 (oxidized culture strain ARC 03, isolated from sample no. 3935, the Gulf of Ob).

***Stephanodiscus. hantzschii* Grunow in Cleve & Grunow (strain ARC05)**

**Figure 3:**
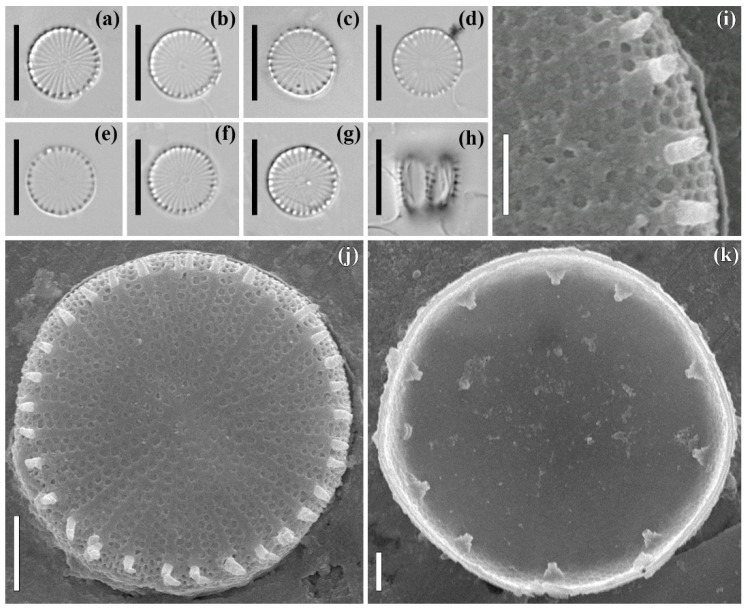
*Stephanodiscus. hantzschii* Grunow in Cleve & Grunow. Strain ARC05. Slide no. 08442. LM, DIC: valve face (**a**–**g**), valve mantle (**h**). SEM: (**i**,**j**) external view, (**k**) internal view. (**a**–**i**) size diminution series, (**i**) fragment of valve, (**j**,**k**) general view. Scale bars: 10 μm (**a**–**h**), 2 μm (**j**), 1 μm (**k**).

**The LM** images are shown in Figure 3a–h. The frustules are tied with well visible spines. The valve mantle is rather high (Figure 3h). The valves are circular with a flat valve face. The diameter is 8.9–9.6 μm. The striae are radiate, fascicles in circumferential count 10–12 in 10 μm.

**The SEM**, external views are shown in Figure 3i,j. A central annulus is present. Central outgrowths are absent (Figure 3i). The striae are uniseriate at the valve center, becoming tri-seriate near the valve mantle (Figure 3i,j). The areolae number 35–37 in 10 μm (in one row). There are spines between each fascicle, positioned at the valve face/mantle junction (Figure 3i,g). The fultoportulae are open with tubes located under the spines (Figure 3j).

**The SEM**, internal view is shown in Figure 3k. The valve surface is flat. The fultoportulae are well developed, and their tubes have three satellite pores. The only rimoportula is located on the valve mantle. The ripomortula lips are well expressed. The specimens shown are slide no. 08442, stub no. ARC 05 (oxidized material of culture strain ARC 05, isolated in sample no. 3935 from the Gulf of Ob).

#### 2.1.2. Green Algae

***Desmodesmus armatus* (Chodat) Hegewald (strain ARC06)**

**Figure 4:**
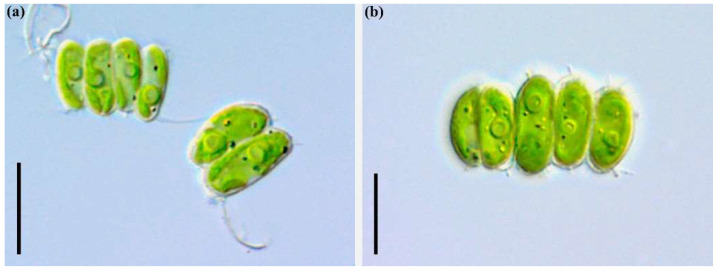
*Desmodesmus. armatus* (Chodat) Hegewald. Strain ARC06. Coenobia with different number of cells (**a**,**b**). Scale bars: 10 μm.

**The LM** images are shown in Figure 4a,b. Coenobia of 2 or 4(–8) linearly to slightly alternately cells. The cells are 8.2–12.3 µm long, 2.8–4.6 µm wide, ellipsoid, elongate-ovoid to cylindrical in shape. The outermost wall of the marginal cells is slightly convex. The apices are rounded and diagonally symmetrical, with an oblique tooth, sometimes on one apex of each cell, carrying main spines on only one apex of each marginal cell. The cell wall has a longitudinal ridge. The chloroplast is parietal with a single pyrenoid.

#### 2.1.3. Yellow-Green Algae

***Tribonema* cf. *minus* (Wille) Hazen (strain ARC10)**

**Figure 5:**
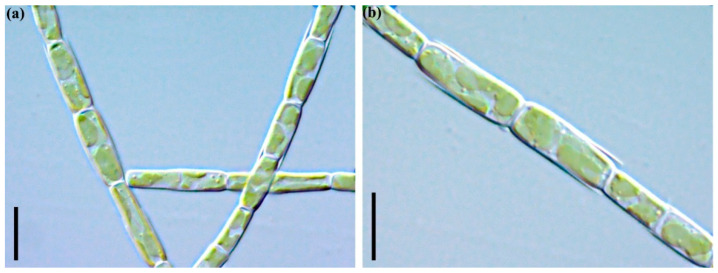
*Tribonema.* cf. *minus* (Wille) Hazen. Strain ARC10. Filaments—(**a**,**b**). Scale bars: 10 μm.

The LM images are shown in Figure 5a,b. The cells are cylindrical, 4.6–5.4 µm wide, 11.1–15.1 µm long, with a length to width ratio of 2.4–2.8. The chloroplasts number 2–4, are relatively large and disc-like, and are arranged almost symmetrically within the cell.

### 2.2. Molecular Description of the Algae Strains

#### 2.2.1. Diatoms

**Figure 6:**
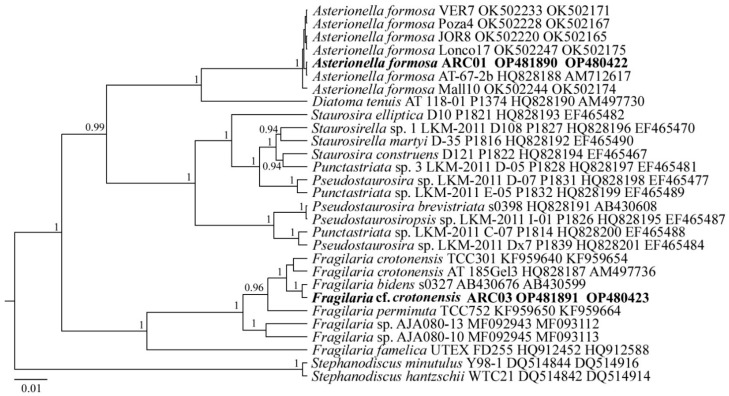
Phylogenetic position of strains *Asterionella formosa* ARC01 and *Fragilaria* cf. *crotonensis* ARC03 (indicated in bold) based on Bayesian inference for the partial 18S rRNA and *rbc*L genes. Total length of the alignment is 1446 characters. Posterior probabilities exceeding 0.9 of BI (constructed by Beast) are presented in order on the nodes. Strain numbers (if available) and GenBank numbers are indicated for all sequences.

The reads of the ARC01 and ARC03 strains were included in the alignments along with corresponding sequences of 27 diatom species, downloaded from GenBank, for the phylogeny reconstruction of pennate diatoms (the taxa names and Accession Numbers are given in Figure 6. The centric diatoms of the genus *Stephanodiscus* were set as the outgroups.

***Asterionella formosa* ARC01**

Sequence data: The partial 18S rRNA gene sequence comprises the V4 domain (GenBank accession number OP480422) and a partial *rbc*L sequence (GenBank accession number OP481890) for the strain *A. formosa* ARC01.

Molecular analysis: In the phylogenetic tree for pennate diatoms based on the *rbc*L and 18S rRNA genes, the strain *A. formosa* ARC01 forms a common clade with all *A. formosa* strains with the highest statistical support; the posterior probability (PP) is equal to 1.0 (Figure 6).

***Fragilaria* cf. *crotonensis* ARC03**

Sequence data: The partial 18S rRNA gene sequence comprises the V4 domain (GenBank accession number (further on, GenBank) OP480423) and a partial *rbc*L sequence (GenBank OP481891) for the strain *F.* cf. *crotonensis* ARC03.

Molecular analysis: In the phylogenetic tree based on the *rbc*L and 18S *r*RNA genes, the *F.* cf. *crotonensis* ARC03 strain forms a common clade with Fragilaria species with the highest statistical support (PP = 1.0) (Figure 6). In this case, the strains ARC03 and *F. bidens* s0327 form a separate subclade, slightly isolated from the clade with *F. crotonensis* strains.

***Stephanodiscus hantzschii* ARC05**

**Figure 7:**
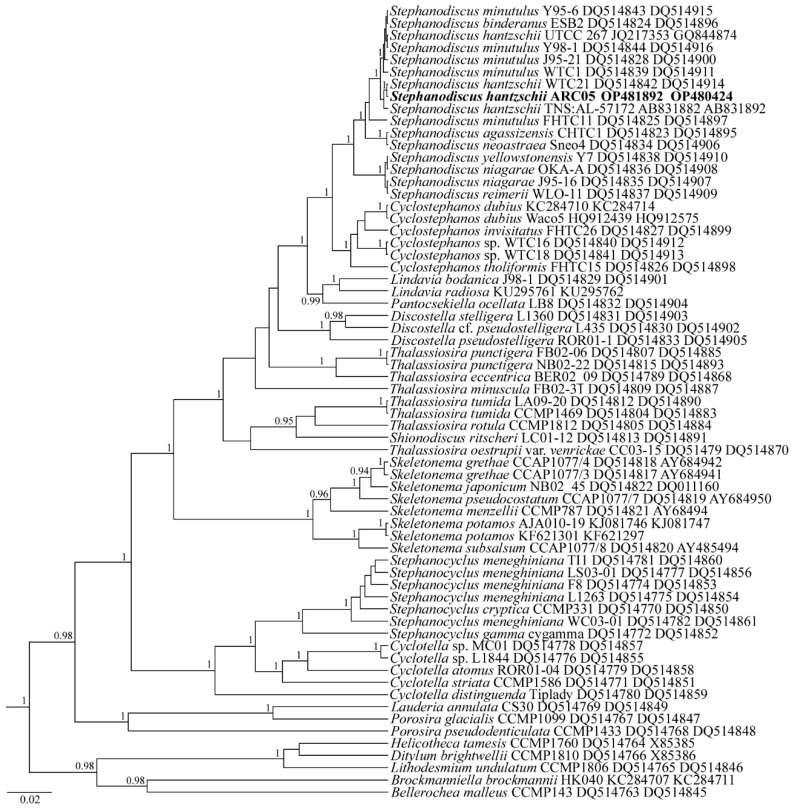
Phylogenetic position of the strain *Stepanodiscus hantzschii* ARC05 (indicated in bold) based on Bayesian inference for the partial 18S rRNA and *rbc*L genes. Total length of the alignment is 1408 characters. Posterior probabilities exceeding 0.9 of BI (constructed by Beast) are presented in order on the nodes. Strain numbers (if available) and GenBank numbers are indicated for all sequences.

For the phylogeny reconstruction of centric diatom, the reads of the ARC05 strain were included in the alignments with sequences of 64 diatom species downloaded from GenBank (the taxa names and Accession Numbers are given in Figure 7). Diatoms from the genera *Bellerochea*, *Brockmanniella*, *Ditylum*, *Helicotheca,* and *Lithodesmium* were set as the outgroup.

Sequence data: The partial 18S *r*RNA gene sequence comprises the V4 domain (GenBank OP480424) and a partial *rbc*L sequence (GenBank OP481892) for the strain *S. hantzschii* ARC05.

Molecular analysis: In the phylogenetic tree based on the *rbc*L and 18S *r*RNA genes, the *S. hantzschii* ARC05 strain forms a common clade with the strain *S. hantzschii* WTC21 with the highest statistical support (PP = 1.0) (Figure 7). All the *Stephanodiscus* strains form a monophyletic clade with the maximum statistical support (PP = 1.0).

#### 2.2.2. Green Algae

**Figure 8:**
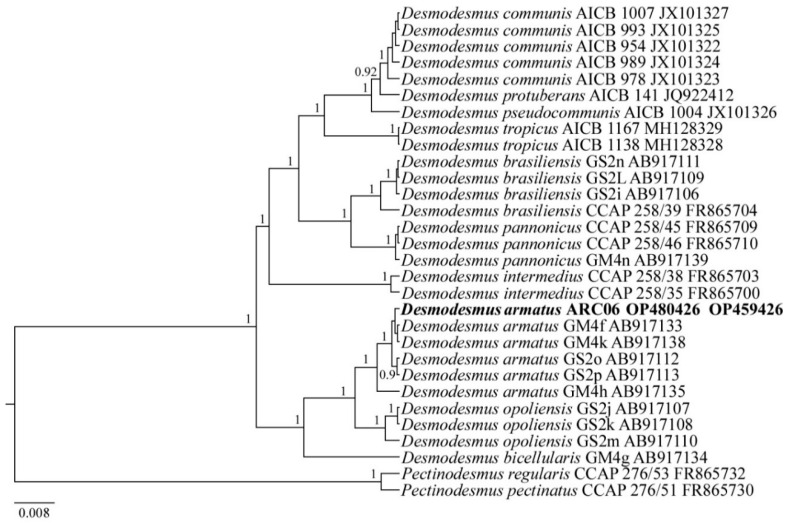
Phylogenetic position of the strain *Desmodesmus armatus* ARC06 (indicated in bold) based on Bayesian inference for the partial 18S rRNA gene and ITS1–5.8S rDNA–ITS2 region. Total length of the alignment is 2132 characters. Posterior probabilities exceeding 0.9 of BI (constructed by Beast) are presented in order on the nodes. Strain numbers (if available) and GenBank numbers are indicated for all sequences.

The reads of the ARC06 strain were included in the alignments with sequences of 29 green algae species, downloaded from GenBank, for the phylogeny reconstruction of Chlorophyceae (the taxa names and Accession Numbers are given in Figure 8). Chlorophycean species from the genus *Pectinodesmus* were set as the outgroup.

***Desmodesmus armatus* ARC06**

The sequence data include the partial 18S rRNA gene sequence (GenBank OP480425) and ITS1–5.8S rDNA–ITS2 region sequence (GenBank OP459426) for the strain *D. armatus* ARC06.

Molecular analysis: In the phylogenetic tree based on the 18S rRNA gene and ITS1–5.8S rDNA–ITS2 region, all *Desmodesmus* species formed two separate clades (Figure 8). *D. armatus* ARC06 joins this lineage, which includes *D. armatus, D. bicellularis* and *D. opoliensis* strains, with the highest support. Within this clade, the new strain is included in the subclade of *D. armatus* with the statistical support PP 1.0.

#### 2.2.3. Yellow-Green Algae

**Figure 9:**
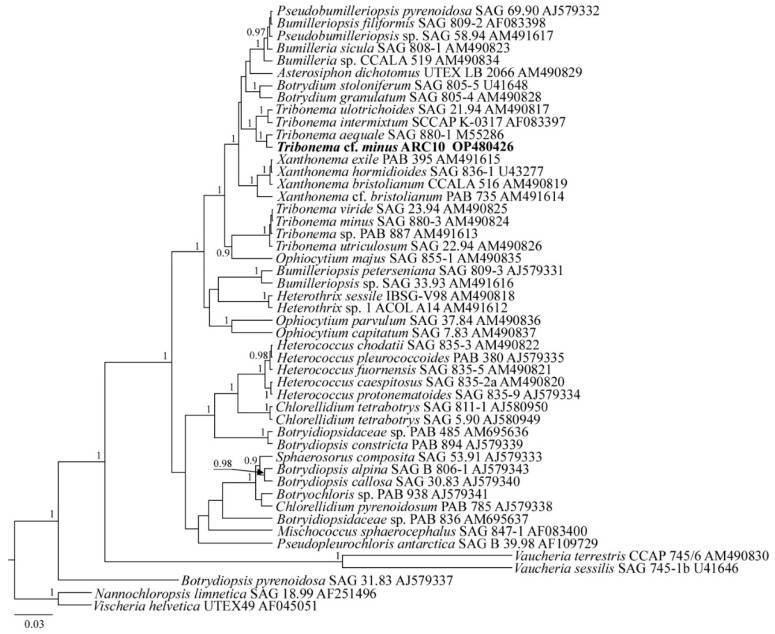
Phylogenetic position of the strain *Tribonema* cf. *minus* ARC10 (indicated in bold) based on Bayesian inference for the partial 18S rRNA gene. Total length of the alignment is 1041 characters. Posterior probabilities exceeding 0.9 of BI (constructed by Beast) are presented in order on the nodes. Strain numbers (if available) and GenBank numbers are indicated for all sequences.

The read of the strain ARC10 was included in the alignment along with the corresponding sequences of 48 Xanthophyceae and Eustigmatophyceae species downloaded from GenBank (the taxa names and Accession Numbers are given in Figure 9). The Eustigmatophycean species *Nannochloropsis limnetica* and *Vischeria helvetica* were set as the outgroups.

***Tribonema* cf. *minus* ARC10**

The sequence data include the partial 18S rRNA gene sequence (GenBank OP480426) for the strain *T.* cf. *minus* ARC10.

Molecular analysis: In the phylogenetic tree based on the 18S rRNA gene, all the *Tribonema* strains form two separate clades (Figure 9). The first (basal) subclade is represented by strains from the species *T. minus*, *T. utriculosum,* and *T. viride*. The second subclade (terminate) is formed by strains of *T. aequale*, *T. intermixtum*, and *T. ulotrichoides* which include ARC10 (PP = 1.0). *T.* cf. *minus* ARC10 occupies a sister position to the strain *T. aequale* SAG 880-1 (PP = 1.0).

### 2.3. FDOM Fluorescence

#### 2.3.1. PARAFAC Components

**Figure 10:**
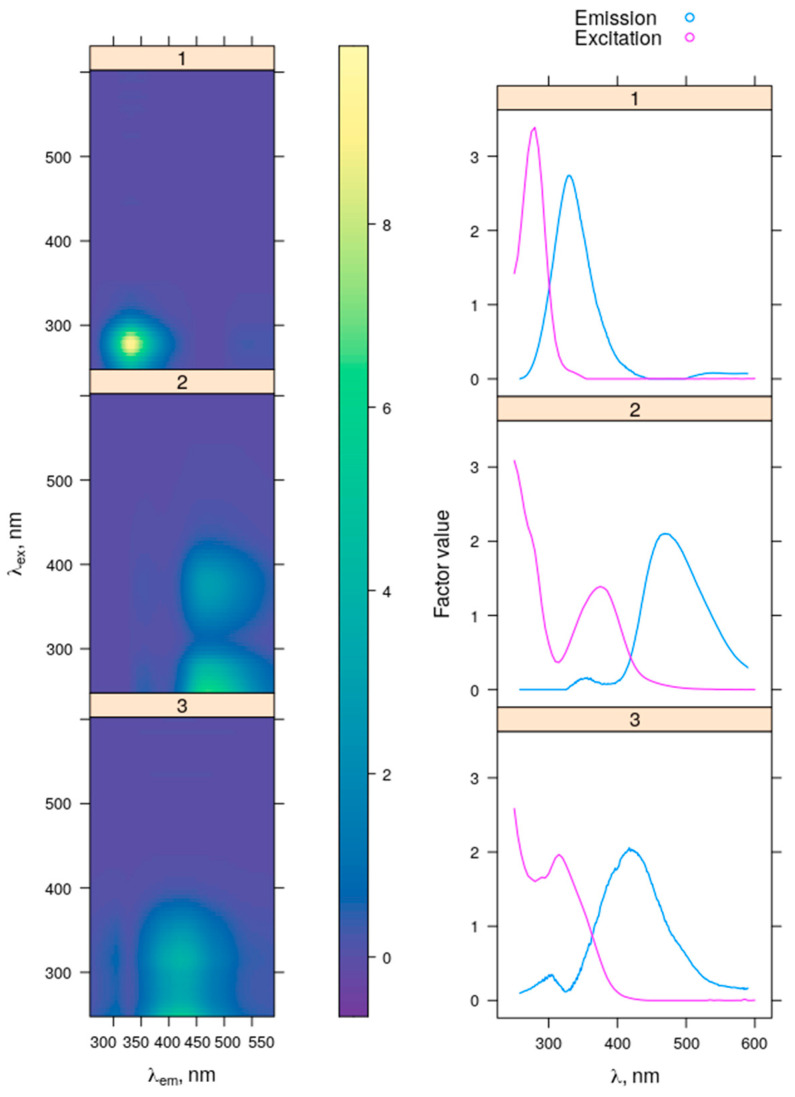
EEMs (**left panel**), excitation and emission spectra (**right panel**) for each identified PARAFAC component.

Three fluorescent components were identified by PARAFAC decomposition. All of them had one pronounced emission peak with one or two excitation maxima (Figure 10). PARAFAC components with spectral characteristics similar to that obtained in our study are presented sufficiently in the OpenFluor database [39].

Component C1 (λ_ex_ = 278 nm; λ_em_ = 330 nm) exhibited protein-like fluorescence typical for the conventional T fluorophore [40,41]. Both the 20-nm shift of the C1 fluorescence maximum towards shorter wavelengths from the fluorescence maxi-mum of pure tryptophan amino acid solution (λex = 275 nm; λem = 350 nm) and the lack of pronounced tyrosine-like fluorescence suggests the presence of proteins in the studied water samples [11,42]. Component C2 (λ_ex_ ≤ 300 nm, 376 nm; λ_em_ = 470 nm) is usually assigned to the ubiquitous humic-like component typical for high molecular weight humic compounds [43,44] of terrestrial origin [45,46]. C2 has been also reported to be derived from microbial metabolism [47,48]. Component C3 (λ_ex_ ≤ 260 nm, 315 nm; λ_em_ = 421 nm) is characterized by humic-like fluorescence similar to Coble’s peak A [40]. It is abundant mostly in freshwater bodies (rivers, lakes) and in estuarine, coastal and shelf regions of the oceans, corresponding usually to the high impact of terrestrial humic-like DOM [49,50].

#### 2.3.2. FDOM Production

At the beginning of the experiment, the fluorescence intensity of C1–C3 PARAFAC components in the WC liquid medium [51] and the Bold basal medium (BBM) [52], which are used for cultivation of microalgae strains (see Section 5.1 for details), did not exceed 0.006 R.U., 0.004 R.U., and 0.008 R.U. for the C1, C2, and C3, respectively (Table 1). A considerable increase in CDOM content was observed by the end of algae cultivation. Thus, at the stationary phase of algae growth, the fluorescence intensity of individual components accounted for 0.146 ± 0.008 R.U. (C1), 0.108 ± 0.009 R.U. (C2), and 0.086 ± 0.002 R.U. (C3). The largest CDOM concentrations were typical for the green (ARC06) and yellow green (ARC10) strains. Assuming that FDOM of pure media was not consumed during cultivation, the contribution of the media FDOM fluorescence to the total fluorescence signal at the end of the experiment was estimated as 3.7%, 2.8%, and 9.1% for the C1, C2, and C3, respectively.

The spectral characteristics of the fluorescent components obtained from EEM + PARAFAC decomposition are presented in Table 2; these data enable detailed comparisons with previous studies. Our results are in a good agreement with the data obtained when analyzing FDOM dynamics during the growth of green algae *Chlorella vulgaris* [26]. In the study by Ly et al. [26], the component with emission maximum at 420 nm has been identified as microbial fulvic-like strains [53,54], and the component with an emission maximum located at longer wavelengths of 450 nm has been assigned to microbial humic-like strains, based on earlier studies [55]. It is suggested that increasing of content of the protein-like component during algae cultivation could be driven by the release of intracellular algogenic organic matter substances. Furthermore, the spectral characteristics of CDOM at the stationary phase of algae growth are found to be consistent with those of the CDOM of black waters in Lake Taihu [32], except for the component exhibiting tyrozine-like fluorescence (peak B).

The fluorescent components presented by Zhang et al. [29,30] and Chen et al. [31] differ from our results in two ways: firstly, by the shift of the protein-like fluorescence maximum to shorter wavelengths, so that it has been identified as a tyrozine-like (peak B); and secondly, by the presence of an additional component characterized by marine humic fluorescence (peak M) [29,30,31].

**Figure 11:**
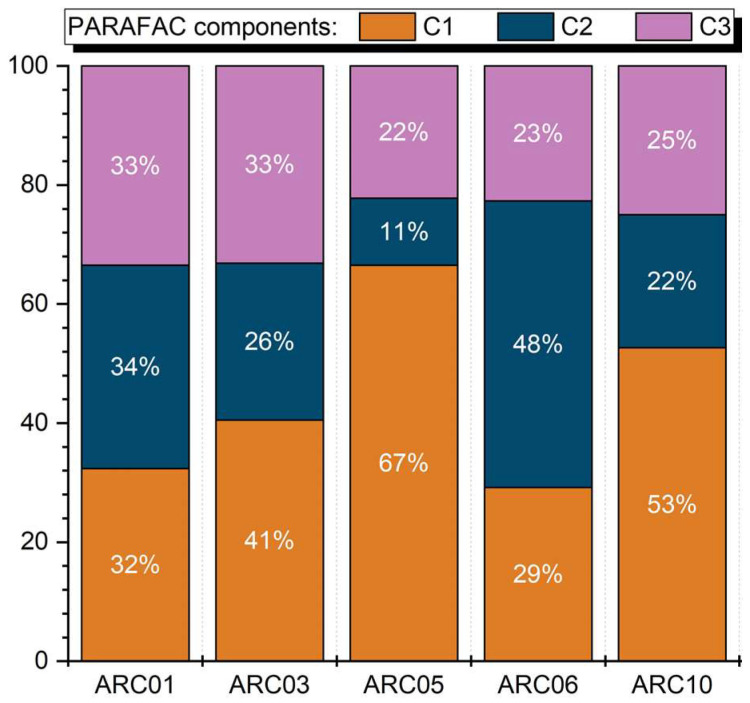
Contribution of C1–C3 PARAFAC components to the total FDOM fluorescence intensity for the different strains of microalgae at the stationary phase of growth.

Nowadays, PARAFAC decomposition of FDOM EEMs are widely used in the studies of biogeochemical processes at high latitudes; see, for example, [56,57,58,59]. The protein-like fluorescence is considered as a marker of autochthonous DOM, whereas humic-like fluorescence is usually interpreted as an indicator of terrestrially-derived DOM, formed in soils by the decay of dead plants. Thus, the large amount of algogenic FDOM exhibiting humic-like fluorescence can lead to overestimation of the vascular plant inputs. Based on the fluorescence intensity of the C1–C3 components, given in Table 1, we estimated the contribution of the fluorescence intensity of single components to the total fluorescence, which can be important for a better evaluation of DOM sources from the EEM + PARAFAC analysis. At the end of cultivation of the different microalgae strains, the fraction of CDOM fluorescent components C1–C3 in the samples ranged from 11% to 67%. The protein-like fluorescence (C1) prevailed in most cases, and the average percentage was 45 ± 16%. It varied between 29% and 67% with the maximum contribution to the total fluorescence intensity observed in the case of the diatom strain ARC05 from the Gulf of Ob (Figure 11). The only exception was the strain of green algae from the Yenisei Gulf (ARC06), in which a strong predominance of humic-like fluorescence (C2) of 48% was observed. The average portion of C2 was found to be 28 ± 14%. The contribution of C3 fluorescence was quite similar for all the algae strains, ranging from 22 to 33%. Its average fraction was comparable to that of C2 and estimated as 27 ± 5%.

## 3. Discussion

There exists a wide range of FDOM sources and sinks in the Arctic Ocean, and different FDOM components are associated with different processes and source waters. The evaluation of FDOM and its components is considered to have a significant potential for improving our understanding of biogeochemical cycling [60]. The study of algae-derived FDOM is essential for the determination the contribution of microalgae to the FDOM production and balance of aquatic ecosystems [26].

Our results demonstrate that FDOM released from algae of the Ob and Yenisei gulfs exhibit both protein-like (peak T) and humic-like (peaks A and C) fluorescence. Some portion of newly produced DOM is labile, thus representing an essential component of microbial food webs. The microbial degradation is a potentially significant decomposition mechanism for this DOM fraction. Another portion of DOM is more recalcitrant [61]. It may be considered a tracer for water mass circulation in the Arctic Ocean. The production of both protein-like and humic-like FDOM is consistent with previous studies of DOM released during algae growth. For example, it is reported that the protein-like and humic-like fluorophores are produced by four phytoplankton species of the genera *Chaetoceros*, *Skeletonema*, *Prorocentrum*, and *Micromonas* [28]. Similarly, protein- and humic-like fluorescence has been measured in DOM extracted from three common species of bloom-forming algae (*Alexandrium tamarense*, *Chaetoceros affinis,* and *Microcystis* sp.) [55]. A direct comparison of the spectral characteristics of individual fluorescence components provided in the abovementioned studies is complicated, since the traditional “peak picking” technique has been applied in the EEMs analysis. In this case, the overlapping of absorption bands results in maxima displacing (from true maxima); therefore, this may hinder the visual localization of different fluorescence maxima in complex mixtures [46].

Comparison of the optical characteristics of the obtained PARAFAC components with the results of previous studies (Table 2) shows the high level of variability in algae-derived FDOM quality. Differences are observed both in the number of fluorescent components and in the position of their emission maxima. For example, components presented by Zhang et al. [29,30] and Chen et al. [31] included additional fluorophore characterized by marine humic fluorescence (peak M), and protein-like fluorescence was blue-shifted compared with our result [29,30,31]. Such variation is most likely the result of a number of factors that affect the algae metabolism. Indeed, the composition of algae-derived DOM is complex and aggregates different classes of chemical compounds: proteins, polysaccharides, etc. Its content and molecular composition depend considerably on species composition, as well as on the physical and chemical properties of the environment, such as the temperature, light regime and nutrient availability [62,63]. For example, the increase in temperature during the cultivation of green algae can lead to an increase in the molecular weight distribution of DOM and a change in the ratio of proteins and carbohydrates released into the environment by algae cells [64]. Additionally, the contribution of proteins and polysaccharides depends on abundance and biolability of nutrients. It has been demonstrated that a decrease in the N/P ratio leads to an increase in the portion of proteins and a higher degree of hydrophilicity in the DOM fraction [65].

In the present study, the temperature and light intensity were identical during cultivation of all the microalgae strains, and the media were chosen to provide sufficient nutrient supply for all species. Therefore, differences in the quality of DOM released during cultivation of algae described here are most likely related to the peculiarities of metabolism of various strains, not to the environmental conditions. The difference in the FDOM quality at the stationary phase of algae growth was revealed by assessing the contribution of the C1–C3 PARAFAC component fluorescence relative to the total FDOM fluorescence of the samples. The portion of C3 component (peak A) showed little variation and the smallest percentage (27 ± 5%). Protein-like fluorescence (C1) was most intense (45 ± 16%), but along with humic-like fluorescence with emission maximum at 470 nm (C2, 28 ± 14%) varied considerably for different algae strains. Thus, the intensity ratio C1/C2 ranged from 0.6 to 5.9.

In the recent study of Drozdova et al. [66,67], the distribution of chlorophyll-a CDOM were analyzed in the river-influenced areas of Siberian shelf seas. The maximal chlorophyll-a concentrations were typical for the river plumes (salinity < 27). River plume waters are also found to exhibit intense protein-like and humic-like fluorescence. Direct optical investigation of FDOM, produced during the cultivation of the studied microalgae strains, showed that algogenic FDOM can contribute to both protein-like and humic-like fluorescence.

## 4. Study Area and Sampling

**Figure 12:**
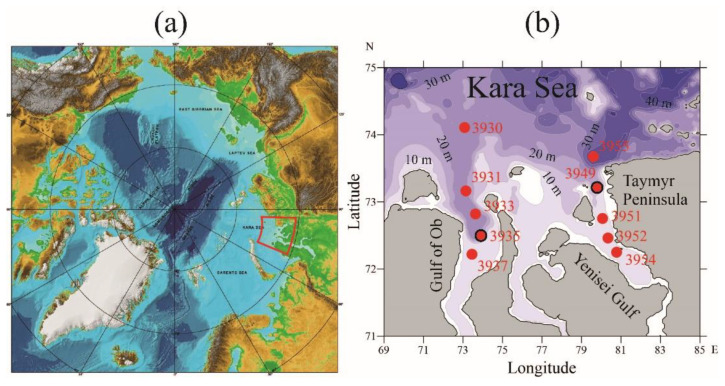
(**a**) IBCAO map of the Arctic Ocean; the red contour indicates the area of the field survey performed in the Kara Sea. (**b**) Bathymetry of the southern part of the Kara Sea; red circles indicate the locations of vertical thermohaline profiling; red circles with black contours indicate locations of water sampling in the Gulf of Ob (station no. 3935) and the Yenisei Gulf (station no. 3949).

The Gulf of Ob and the Yenisei Gulf are located in the southern part of the Kara Sea in the Arctic Ocean (Figure 12). These gulfs are among the largest estuaries in the world ocean. The Gulf of Ob is twice as long (850 km) as the Yenisei Gulf (400 km); both gulfs are rather narrow (30–80 km). The distance between the gulfs is 200 km.

The Ob and Yenisei gulfs receive approximately 530 and 630 km^3^ of annual freshwater discharge, respectively, which accounts for one third of the total freshwater runoff to the Arctic Ocean [68] and ~3% of the total freshwater runoff to the world ocean [69]. These gulfs have different hydrologic regimes due to differences in seasonal variability of the Ob and Yenisei rivers’ discharges [70].

Discharges from the Ob and Yenisei gulfs form a large Ob-Yenisei plume, which covers 200,000–250,000 km^2^ in the Kara Sea [71,72,73,74,75]. This river plume predetermines the water structure and circulation at the shelf of the Kara Sea and affects local ecosystems strongly [76,77].

### 4.1. Environmental Conditions

**Figure 13:**
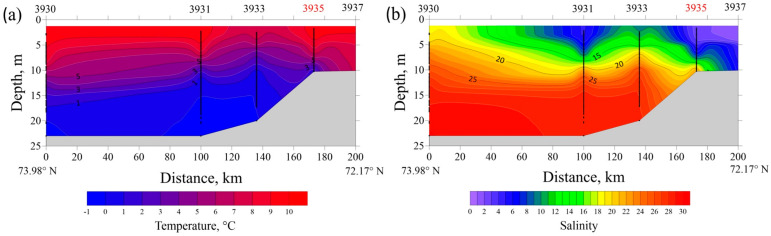
The vertical temperature (**a**) and salinity (**b**) structure along the transect in the Gulf of Ob (15–16 August 2021). Water samples analyzed in this study were collected at station no. 3935 (indicated by red).

**Figure 14:**
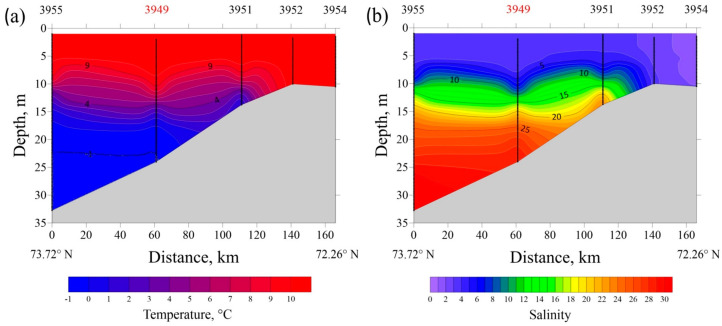
The vertical temperature (**a**) and salinity (**b**) structure along the transect in the Yenisei Gulf (17–19 August 2021). Water samples analyzed in this study were collected at station no. 3949 (indicated by red).

Vertical thermohaline profiles at transects in the Ob and Yenisei gulfs in August 2021 are presented at Figure 13 and Figure 14. A typical bilayer structure is observed in both estuaries with a low-saline (<15) and warm (>7 °C) surface layer and a saline (>25) and cold (<3 °C) bottom layer with sharp vertical gradients at their interface. The southernmost stations at both transects are located in shallow areas (10–12 m deep) affected greatly by river discharge, so water salinity ranges from 0 to 2 from surface to bottom.

The resulting two-layered structure is stable in the Yenisei Gulf, where a homogenous surface layer (from surface down to 5–6 m depth) with salinity of 3–4 and temperature of 10–11 °C is formed (Figure 14). Along the transect in the Yenisei Gulf, near-bottom water salinity increases steadily from 23 at 15 m depth (station no. 3951) to 31 at 32 m depth (station no. 3955), while the corresponding bottom temperatures decreased from 2 to −1 °C. Compared with the Yenisei Gulf, the vertical thermohaline structure is more variable along the transect in the Gulf of Ob, due to meandering of the main seaward flow of freshened water within the estuary (Figure 13). In the Gulf of Ob, a mixed surface layer in the central part of the northward flow of freshened water is 5–6 m deep (salinity of 5–7 and temperature of 9–11 °C), which is similar to that in the Yenisei Gulf.

### 4.2. Field Work

Sampling was carried out in the Ob and Yenisei gulfs during the 58th cruise of R/V “Akademik Ioffe” on 15–19 August 2021, organized by the “Arctic Floating University” (Russian Scientific and Educational Program) (Figure 12). The measurements were performed at 10 stations organized into two longitudinal transects in the northern parts of the gulfs. The vertical thermohaline structure was obtained using a CTD probe (SBE 911plus) at a 24 Hz sampling rate. This CTD probe was equipped with two parallel temperature and conductivity sensors; the mean difference did not exceed 0.01 °C for temperature and 0.005 PSU for salinity. The CTD-data were processed by standard programming package (SBE Data Processing, version 7.26.7) using the recommended settings.

Samples were collected in the upper 0.5–1.0 m water layer using the Rosette-based sampling system with mounted plastic Niskin bottles at station no. 3935 in the Gulf of Ob and station no. 3949 in the Yenisei Gulf (Figure 12b: red circles with black contours). After sampling, the water was placed in sterile polypropylene containers and kept cool in a bright place until microalgae strains were isolated in the laboratory. In this article, we used five strains of microalgae, including three isolated from the Gulf of Ob (ARC01, ARC03, and ARC05) and two from the Yenisei Gulf (ARC06 and ARC10).

## 5. Materials and Methods

### 5.1. Algae Strains’ Isolating and Cultivation Conditions

Monoclonal microalgae strains were established by targeted micro-pipetting of cells under an inverted microscope. The single algae cells from water samples were transferred by glass micropipette to a glass slide and rinsed several times using sterile water to remove as many bacteria as possible. Then the cells were transferred by a new glass micropipette into immunology microplates with nutrient medium. Thereafter, the plates were wrapped with Parafilm^®^ and stored at 22–25 °C in a growth chamber with a 16:8 h light/dark photoperiod. After 10–14 days, single colonies composed of algae cells, which have not been contaminated with fungi and yeast, were picked and carefully transferred to a Petri dish with sterile nutrient medium. The dishes were wrapped with Parafilm^®^ and stored at 8–10 °C in a climate chamber at the same photoperiod of 16:8 [78]. The inoculums of the diatom strains (ARC01, ARC03, and ARC05) were maintained in WC liquid medium [51], and the strains of green (ARC06) and yellow-green (ARC10) algae were maintained in Bold basal medium (BBM) [52] prior to the experiments.

Before the experiments, the culture medium was autoclaved for sterilization. The pH of the medium was adjusted to 7.0–7.2. For the cultivation experiments, the cells of the microalgae strains were grown in complete WC and BBM medium in 300 mL glass flasks containing 150 mL culture media. For this, 1 mL inoculum of each strain was added to a flask. The initial cell concentration in the medium was 10^5^ cells mL^−1^. The flasks were incubated under white fluorescent light illumination at 100 µmol PAR photons m^−2^ s^−1^ at a 16:8 h light/dark photoperiod with gentle shaking at 150 rpm. For all the experimental runs, the temperature was maintained at 25 ± 0.5 °C. The cultivation of each microalgae strain was carried out in triplicate. The algae were cultivated until reaching the stationary phase of growth.

### 5.2. Molecular Analyses

Total DNA from the studied strains was extracted using Chelex 100 Chelating Resin, molecular biology grade (Bio-Rad Laboratories, Hercules, CA, USA), according to the manufacturer’s protocol 2.2. Partial 18S rDNA fragments (387–388 bp, including the highly variable V4 region of the 18S rRNA gene) of diatoms were amplified using primers D512for and D978rev [79]. Partial *rbc*L plastid gene fragments (966–1023 bp) were amplified using primers *rbc*L404+ [80] and dp7 [81]. Partial 18S *r*DNA fragments (983–1415 bp) of chlorophycean and xanthophycean algae were amplified using primers 18S-F [82] and picoR2 [83,84]. Amplification of the 652 bp ITS1–5.8S *r*DNA–ITS2 region for the strain ARC06 was performed using ITS1 and ITS4 primers [85].

Amplifications were carried out using premade polymerase chain reaction (PCR) mastermixes (ScreenMix by Evrogen, Moscow, Russia). Amplification conditions for the V4 region were as follows: initial denaturation for 5 min at 95 °C followed by 35 cycles of 30 s denaturation at 94 °C, 30 s annealing at 52 °C, and 50 s extension at 72 °C, with the final extension for 10 min at 72 °C. Amplification conditions for the *rbc*L gene were as follows: initial denaturation for 5 min at 95 °C followed by 45 cycles of 30 s denaturation at 94 °C, 30 s annealing at 59 °C, and 80 s extension at 72 °C, with the final extension for 10 min at 72 °C. Amplification conditions for the 18S rRNA gene were as follows: initial denaturation for 5 min at 95 °C followed by 40 cycles of 30 s denaturation at 94 °C, 40 s annealing at 50 °C, and 2 min extension at 72 °C, with the final extension for 5 min at 72 °C. Amplification conditions for the ITS1–5.8S rDNA–ITS2 region were as follows: initial denaturation for 5 min at 95 °C followed by 35 cycles of 30 s denaturation at 94 °C, 30 s annealing at 60 °C, and 60 s extension at 72 °C, with the final extension for 5 min at 72 °C. PCR products were visualized by horizontal electrophoresis in 1.0% agarose gel stained with SYBRTM Safe (Life Technologies, Waltham, MA, USA). The products were purified with a mixture of FastAP, 10 × FastAP Buffer, Exonuclease I (Thermo Fisher Scientific, Waltham, MA, USA) in aqueous solution. Sequencing was performed using a Genetic Analyzer 3500 instrument (Applied Biosystems, St. Louis, MO, USA).

Editing and assembling of the consensus sequences were carried out by processing the direct and reverse chromatograms in Ridom TraceEdit (ver. 1.1.0) (Ridom GmbH, Münster, Germany) and Mega7 software [86]. The nucleotide sequences of the 18S rRNA and *rbc*L genes were aligned separately using the Mafft v7 software and the E-INS-i model [87]. The final alignments were carried out by a procedure whereby unpaired sites were visually determined and removed from the beginning and the end of the resulting matrices. For the protein-coding sequences of the *rbc*L gene, it was checked if the beginning of the aligned matrix corresponded to the first position of the codon (triplet). The resulting alignments had lengths of 394 (18S rDNA for centric diatoms), 417 (18S rDNA for pennate diatoms), 1014 (*rbc*L for centric diatoms), 1029 (*rbc*L for pennate diatoms), 2132 (18S rDNA and ITS1–5.8S rDNA–ITS2 region for chlorophycean algae), and 1041 (18S rDNA for xanthophycean algae) characters. After removal of the unpaired regions, the aligned 18S rDNA gene sequences were combined with the *rbc*L gene or ITS1–5.8S rDNA–ITS2 region sequences into a single matrix Mega7.

The data set was analyzed using the Bayesian inference (BI) method implemented in Beast ver. 1.10.1 software [88] to construct a phylogeny. For the alignment partition (most appropriate substitution model), shape parameter α and a proportion of invariable sites (pinvar) were estimated using the Bayesian information criterion (BIC) as implemented in jModelTest 2.1.10 [89]. This BIC-based model selection procedure selected the following models, shape parameter α and a proportion of invariable sites (pinvar) for the centric diatoms tree: TrN + I + G, α = 0.6810 and pinvar = 0.4210 for 18S rDNA; HKY + I + G, α = 0.5180 and pinvar = 0.7140 for the first codon position of the *rbc*L gene; TPM1 + I + G, α = 0.8250 and pinvar = 0.8270 for the second codon position of the *rbc*L gene; TVM + I + G, α = 1.2400 and pinvar = 0.1490 for the third codon position of the *rbc*L gene. This BIC-based model selection procedure allowed the selection of models, shape parameter α, and pinvar for the pennate diatoms tree: TIM2 + G and α = 0.2630 for 18S rDNA; F81 + I and pinvar = 0.8360 for the first codon position of the *rbc*L gene; JC + I and pinvar = 0.9170 for the second codon position of the *rbc*L gene; TPM3uf + I and pinvar = 0.3920 for the third codon position of the *rbc*L gene. This BIC-based model selection procedure selected the TrNef + I + G model, α = 0.6880 and pinvar = 0.6260 for 18S rDNA and ITS1–5.8S rDNA–ITS2 tree in chlorophycean algae; TIM2ef + I + G model, α = 0.4550 and pinvar = 0.4640 for the 18S rDNA tree in xanthophycean algae. A Yule process tree was used prior as a speciation model for all phylogenetic reconstructions. The analysis ran for 5 million generations with chain sampling every 1000 generations. The parameters-estimated convergence, effective sample size (ESS), and burn-in period were checked using the Tracer ver. 1.7.1 software (MCMC Trace Analysis Tool, Edinburgh, United Kingdom) [88]. The initial 25% of the trees were removed, and the rest were retained to reconstruct a final phylogeny. FigTree ver. 1.4.4 (University of Edinburgh, Edinburgh, UK) and Adobe Photoshop CC (19.0) software (Adobe, San Jose, CA, USA) were used for visualizing and editing the trees.

### 5.3. Preparation of Slides and Microscopic Analysis

The diatom strains were boiled in H_2_O_2_ (~37%) to dissolve the organic matter and then washed with Milli-Q water four times at 12-h intervals. After decanting and filling with deionized water up to 100 mL, the suspension was spread on to coverslips and dried at room temperature. Permanent diatom slides were mounted in Naphrax^TM^. The light microscopy (LM) observations were performed under a Zeiss Scope A1 microscope (Carl Zeiss Microscopy GmbH, Gottingen, Germany) equipped with an oil immersion objective (100×/n.a.1.4, differential interference contrast) and mounted Zeiss Axio-Cam ERc 5s camera (Carl Zeiss NTS Ltd., Oberkochen, Germany). The ultrastructure was examined under a JSM-6060A (JEOL Ltd., Tokyo, Japan) scanning electron microscope operated at 10 kV and 11-mm distance, in the Laboratory of Structural and Morphological Research, Frumkin Institute of Physical Chemistry and Electrochemistry, Russian Academy of Sciences (Moscow, Russia). For scanning electron microscopy (SEM), suspension aliquots were fixed on aluminum stubs after air-drying. The stubs were sputter coated with 50 nm of gold [90]. The cleaned diatom samples preserved with 96% ethanol are stored at the Laboratory of Molecular Systematics of Aquatic Plants, Timiryazev Institute of Plant Physiology, Russian Academy of Sciences (Moscow, Russia).

Temporary preparations of green and yellow-green algae strains were mounted in Naphrax^TM^. The light microscopic (LM) observations were performed under a Zeiss Scope A1 microscope equipped with an oil immersion objective (100×/n.a.1.4, differential interference contrast) and mounted Zeiss Axio-Cam ERc 5 s camera.

### 5.4. Optical Measurements and PARAFAC Decomposition

The algae strains were cultivated three times in parallel. When the stationary phase of growth was reached, 15 mL of each sample was taken and combined to obtain a sample of 45 mL volume for each single algae strain. The combined samples were then filtered through Whatman 47 mm GF/F fiberglass filters with an effective pore diameter of 0.5–0.7 μm (preliminarily heat-treated at 450 °C) under a 200–400 mbar vacuum using a standard method [91].

The fluorescence and absorbance of DOM were studied with an Aqualog (Horiba Jobin Yvon, Inc., Edison, NJ, USA) system in 1 cm path-length quartz cuvette at room temperature. The spectra were recorded with respect to a blank sample of ultra-pure water at excitation wavelengths varying from 250 to 590 nm with 5 nm increments and emission wavelengths ranging from 260 to 800 nm with 1.12 nm increments. The integration time was set to 2 s. Excitation-emission matrices (EEMs) were corrected for inner filtering effect using the functions provided within the Aqualog software. EEMs were normalized to the area under the water Raman peak (excitation wavelength of 350 nm) of the ultra-pure water sample to produce fluorescence intensities in Raman Units (R.U.).

The dataset consisted of 27 EEMs including the spectra of WC, BBM media, and the filtrate sampled at different cultivation stages. The areas affected by scattering signal were removed and interpolated using Whittaker smoothing [92]: ±18 nm around 1st order Rayleigh and Raman signals, ±30 nm around 2nd order Rayleigh and Raman signals, assuming a Raman shift of 3400 cm^−1^. The widths and the penalty factors were set visually. The area at 18 nm < λ_ex_–λ_em_ < 30 nm was left undefined.

After handling the scattering signal, the resulting spectra were scaled by their standard deviation. Split-half analysis was performed on 32 randomly-set halves, suggesting a three-component model. The PARAFAC model was calculated with nonnegative constraints. All the procedures were performed using the R programming language and the “albatross” package [93].

## Figures and Tables

**Table 1:** Fluorescence intensity (R.U.) of individual FDOM PARAFAC components (C1–C3) in the medium at the beginning of cultivation and at the stationary phase of algae growth.

Algae Strains	C1	C2	C3
ARC01	0.068	0.072	0.070
ARC03	0.045	0.029	0.036
ARC05	0.140	0.024	0.047
ARC06	0.173	0.285	0.134
ARC10	0.303	0.129	0.144
BBM ^1^	0.005	0.002	0.008
WC ^2^	0.006	0.004	0.008

Note. ^1^ BBM (Bold basal medium) is a liquid medium for green and yellow-green algae cultivation; ^2^ WC is a liquid medium for diatom cultivation.

**Table 2:** Excitation and emission maxima of the four fluorescent components identified by PARAFAC modeling for the entire EEM data set, compared with previous PARAFAC models.

Present Study	[29]	[30]	[32]	[31]	[26]	[40]
C1 278/330	-	-	<230(280)/338 240(300)/338	-	270/340	T
-	275/320	275/<300	<230(275)/308	285/322	-	B
C2 < 300(376)/470	265/445	270(365)/453	255(345)/464	350/440 285(395)/497	250(370)/450	C
C3 < 260(315)/421	-	255(330)/412	230/405	-	240(270)/420	A
-	310/380	315/372	-	315/384	-	M

Note. Secondary excitation band is given in brackets.

## Data Availability

Not applicable.
